# Teaching Taste: The TASTE–MED Conceptual Framework for a Multisensory Mediterranean Approach to Food Literacy in Adolescence

**DOI:** 10.3390/nu18040635

**Published:** 2026-02-14

**Authors:** Paula Silva

**Affiliations:** 1 Department of Microscopy, School of Medicine and Biomedical Sciences (ICBAS), University of Porto (U. Porto), Rua Jorge Viterbo Ferreira 228, 4050-313 Porto, Portugal; psilva@icbas.up.pt; 2 iNOVA Media Lab, ICNOVA-NOVA Institute of Communication, NOVA School of Social Sciences and Humanities, Universidade NOVA de Lisboa, 1069-061 Lisbon, Portugal

**Keywords:** health literacy, taste perception, adolescent, education, diet, Mediterranean, schools, food preferences, models, educational

## Abstract

Background/Objectives: Adolescence is pivotal for establishing dietary habits; however, school-based nutritional education remains focused on information dissemination, with minimal effects on behavior modification. Evidence from neuroscience, education, and food literacy indicates that attention, engagement, sensory experiences, and social contexts are integral to effective learning in nutrition education. This article conceptualizes a framework for adolescent food education beyond knowledge transmission, aiming to cultivate taste competence using the Mediterranean Diet as a pedagogical ecosystem. Methods: This study employed a conceptual methodology, utilizing interdisciplinary literature from food literacy, sensory education, developmental neuroscience, educational theory, and public health nutrition. It synthesizes empirical findings and theoretical models to develop the Teaching Autonomous Sensory Taste in the Mediterranean Diet (TASTE–MED) framework. Results: This study introduces taste competence as a multifaceted educational outcome, encompassing sensory, relational, cultural, and reflective dimensions. The TASTE–MED framework outlines how experiential, multisensory, and socially embedded learning processes can be implemented in schools, facilitated by the Mediterranean Diet, which provides a sensory-rich and culturally significant context. The educational implications are discussed in terms of curriculum design, teacher training, family involvement and digital tools. Conclusions: The TASTE–MED framework redefines food literacy as an embodied and socially situated competence rather than a cognitive construct. This framework provides a theoretical foundation for informing the design, evaluation, and research of future interventions, advocating for the transition from information-based nutrition education to competence-oriented food education during adolescence.

## 1. Introduction

Adolescence is a critical developmental period for the formation of long-term dietary behaviors, food preferences, and lifestyle patterns [1,2,3]. During this stage, individuals undergo neurobiological, cognitive, and social changes that influence their processing of food-related information [4,5,6]. Most nutrition education programs for adolescents focus on disseminating information and guidelines, often yielding limited long-term effects on eating behavior [7].

Adherence to the Mediterranean Diet and lifestyle, known for its health and environmental benefits, is declining even in Mediterranean countries. Research shows that less than 25% of people adhere to this lifestyle. Taste aversion, low motivation, time constraints, and reduced social participation are the key barriers [8]. These findings imply that mere awareness and access are inadequate for maintaining healthy and sustainable dietary patterns in older adults. Contemporary food environments are increasingly dominated by ultra-processed foods that are widely available and engineered for palatability, making it difficult to maintain a healthy diet [9,10]. The Mediterranean Diet may be perceived as an idealized model disconnected from adolescents’ food environments. Rather than countering ultra-processed food consumption through information alone, the Teaching Autonomous Sensory Taste in the Mediterranean Diet (TASTE–MED) framework aims to reconstruct food preferences by developing sensory competence, reflective judgment, and self-regulatory capacities that help adolescents critically engage with their food choices.

Recent findings from education, behavioral nutrition, and health promotion indicate that engagement, motivation, and experiential learning are critical components of food literacy interventions among adolescents. Programs incorporating practical activities, social interaction, and self-regulatory skills show increased participation and relevance among adolescents, including those with chronic conditions and disabilities [11,12]. Building on this evidence, this study argues that adolescent food literacy requires a multisensory, experiential, and culturally grounded approach beyond information transmission. Integrating neuroeducation insights on sensory attention and affective engagement in memory, this study proposes a framework centered on Mediterranean food culture that leverages its social and sensory richness to create durable and health-promoting eating habits [13]. TASTE–MED responds to the “ceiling effect” of information-based food education [7,14] by shifting the focus from nutritional knowledge to taste competence as an embodied, socially situated capability that can support durable food literacy in adolescence.

## 2. Why Attention, Engagement, and Learning Matter in Adolescence

### 2.1. Adolescence and Attentional Processes

Adolescence is characterized by the ongoing maturation of the neural systems involved in attention regulation, executive control, and reward processing [15]. During this period, heightened sensitivity to novelty, emotional salience, and social relevance coexists with still-developing cognitive control mechanisms [4,16]. This neurobiological imbalance between an early maturing reward system and a later-maturing prefrontal control system predisposes adolescents to favor immediate stimuli [15,17]. Adolescents preferentially attend to engaging, meaningful, and socially relevant stimuli while showing reduced responsiveness to abstract information [5]. Within food literacy, while traditional education relies on “top-down” cognitive effort to follow health guidelines, adolescents respond better to “bottom-up” sensory experiences. Exclusively theoretical food is less likely to engage dopaminergic reward pathways in the adolescent brain [4]. This developmental profile significantly impacts food education. Content that fails to capture adolescents’ attention will not be encoded effectively. By shifting from nutrient-centrism to multisensory engagement, educators can align with the adolescent brain’s receptivity to rewarding experiences. Thus, attention is a prerequisite for learning rather than a secondary instructional outcome [5,18,19].

### 2.2. Engagement as a Condition for Learning

Studies have shown that adolescent engagement better predicts learning outcomes than content exposure alone. Experiential food education programs demonstrate high attendance, retention, and satisfaction, even among “hard-to-reach” adolescents [12]. A remotely delivered physical and food literacy program for adolescents with intellectual disabilities showed high engagement during guided sessions but lower participation during self-directed practice [11]. These findings indicate that engagement is better facilitated through structured social experiences rather than through isolated tasks. This aligns with adolescents’ needs for social belonging and autonomy [20,21]. Engagement is both a pedagogical tool and a neurobiological necessity for cognitive processing. By involving adolescents in food preparation within Mediterranean commensality, educators use “embodied cognition,” where handling ingredients enhances food literacy internalization [22,23]. Moreover, when these experiences are shared with peers, they transform dietary choices from individual health decisions into shared cultural practices, significantly increasing the likelihood of long-term adherence [24,25].

### 2.3. Motivation and Self-Regulation as Mediating Mechanisms

Evidence indicates that attention alone cannot facilitate meaningful learning. Motivation and self-regulation mediate the relationship between educational exposure and food literacy outcomes [20]. This motivational framework originates in the domestic environment, where the relational climate influences information internalization. Research shows that parental food education enhances children’s food literacy by fostering positive learning motivation rather than merely conveying dietary facts [26]. Parent–child relationship quality provides affective “scaffolding” for motivational states. While families offer an emotional foundation, educational institutions must equip adolescents with cognitive tools for autonomy [27].

School-based interventions combining nutrition education with goal-setting tools have shown sustained improvements in nutritional knowledge and self-regulation [28]. However, these cognitive gains often do not translate to control over emotional eating behaviors, highlighting the limitations of rational engagement.

Cross-national evidence from over 5000 parent–adolescent dyads across ten Arab countries suggests that adolescent literacy may be embedded within familial (il)literacy cycles [29]. By recognizing that food-related learning is a socialized process shaped by family dynamics, institutional quality, and emotional regulation, it becomes clear that Teaching Taste can be understood as more than a classroom exercise, potentially functioning as an intervention within the adolescent’s relational and sensory ecosystem [30].

The disconnect between cognitive knowledge and emotional eating suggests that self-regulation requires more than willpower. By fostering multisensory engagement through “Teaching Taste,” adolescents can develop interoceptive sensitivity. This internal calibration, cultivated within supportive environments, provides a foundation for durable and self-regulated eating patterns [20,22,31].

### 2.4. Social Context and Contemporary Channels of Attention

Adolescents’ attention is increasingly influenced by digital and social environments. Evidence shows that adolescents engaging with social media influencers for health content often report higher health literacy, especially in functional and critical areas [32]. Adolescents selectively engage with food and health information through familiar social channels rather than being disconnected from them. However, higher digital engagement does not lead to better factual knowledge or behavioral changes, highlighting the gap between attention and learning. This indicates the need for education that converts engagement into lasting understanding and preferences [33,34,35].

Recent evidence suggests that adolescents’ engagement with food-related digital content is shaped by their pre-existing food interests. A cross-sectional study of Korean adolescents categorized participants by food-related lifestyles, revealing variations in food literacy, nutrition behaviors, and responses to food media [36]. Adolescents with a high interest in food showed better food literacy and nutrition scores, engaging positively with food media by exploring foods and checking nutrition labels. Those with low food interest, despite similar media exposure, reported more overeating after media consumption and showed lower food literacy and poor dietary habits [36]. No significant differences were found among the groups in dietary moderation, showing that media exposure or interest alone does not ensure self-regulatory eating. This “amplification effect” indicates that digital environments enhance existing motivational tendencies rather than compensate for their absence [37]. Digital literacy alone is insufficient; without foundational sensory interest in food, media exposure may exacerbate poor eating habits by stimulating hedonic consumption without a critical or interoceptive framework [38,39]. Educational interventions must prioritize “sensory priming” and create real-world interest through multisensory exploration to ensure that digital influences are filtered through genuine curiosity and embodied meaning [40].

### 2.5. Limits of Information-Based Food Education

While knowledge acquisition is essential, it alone cannot affect enduring dietary changes during adolescence [7,14]. Studies have shown that awareness of Mediterranean lifestyle principles strongly predicts adherence, although the explanatory power remains limited [8]. The primary barriers include taste aversion, low motivation, and reduced social participation. These findings indicate that adolescent food education may benefit from addressing knowledge, attention, feelings, and relationships with food. This provides a rationale for multisensory, experiential, and socially embedded approaches to food literacy [22,34,41].

Evidence from low-resource contexts highlights the structural limitations of food education when not anchored in sensory familiarity. A study of Venezuelan adolescents showed low recognition of common plant-based foods despite exposure to dietary guidelines [42]. Adolescents cannot engage with nutritional recommendations without a basic sensory and linguistic repertoire for identifying promoted foods. This “bottleneck” reveals a key pedagogical flaw: the assumption that cognitive literacy overcomes sensory unfamiliarity. Without this sensory “alphabet,” dietary guidelines remain cognitively accessible but are behaviorally inoperative [43,44].

Empirical evidence from Mediterranean settings corroborates a “ceiling effect” in information-based education. A Tuscan study showed that while food literacy is linked to healthier preferences, adolescents favored moderately healthy options constrained by taste expectations and habits [45]. The disconnect between cognitive knowledge and emotional eating shows that self-regulation cannot be achieved through willpower or information alone. This requires a shift toward multisensory pedagogy that reestablishes the connection between adolescents’ internal sensory experiences and dietary choices, overcoming attentional biases favoring ultra-processed foods [46,47].

## 3. Experiential and Multisensory Learning in Food Education

### 3.1. From Information to Experience: Operationalizing Experiential Learning in Food Education

The first aspect of operationalization involves establishing conducive learning environments. School-based infrastructure, including food gardens, cooking facilities, and waste management systems, serves as a platform for experiential education in food, nutrition, and sustainability. Evidence from Australian schools shows that this infrastructure is prevalent and valued by educators; however, its pedagogical use remains inconsistent, limited by curricular time, safety concerns, insufficient training, and inadequate prioritization [48]. These findings suggest that experiential learning is not assured by the mere presence of physical resources but rather relies on their deliberate integration into teaching practices.

The second mode involves developing structured experiential curricula that integrate cooking, food systems and nutrition into educational programs. The Teens CAN curriculum exemplifies this approach by combining cooking, agricultural literacy, and nutrition education across modules for adolescents [49]. While evidence on long-term dietary outcomes remains limited, such programs demonstrate how experiential learning can be systematically scaffolded across domains rather than relying on isolated activities.

Studies underscore the importance of sensory engagement as a foundational element of experiential learning. Research in home economics education shows that learning outcomes are linked to the application of taste competencies during cooking activities, whether students follow recipes or engage in creative experimentation [50]. These findings imply that experiential learning can be effectively implemented in both structured and flexible instructional formats if sensory evaluation remains the central component of the activity.

Experiential methodologies employ targeted sensory intervention. Research using visual food models in educational settings shows that brief experiential sessions enhance adolescents’ food knowledge and skills, including sugar literacy and label interpretation, even without immediate behavioral change [51]. Interventions integrating sensory education with repeated exposure to unfamiliar foods have shown increased acceptance and willingness to try novel foods, suggesting that experiential formats can influence food preferences independent of broader dietary modifications.

Experiential learning exists along a continuum of varying intensities, durations, and contextual integrations. Brief experiences, such as food models or sensory sessions, enhance food-related knowledge and skills, while recurrent activities, such as cooking classes, have a greater impact on shaping preferences and food openness. Approaches that integrate experiences across school and home contexts show greater potential to influence food practices, although evidence for long-term behavioral changes remains limited [7,52,53].

Time and repetition are essential for experiential learning in food education. Studies have shown that while short-term interventions enhance knowledge and intentions, they rarely lead to sustained dietary changes. This suggests that experiential learning requires both sensory engagement and temporal continuity for consolidation and integration into routines. Without repetition, experiential activities risk remaining episodic rather than driving lasting, food-related changes [14,54].

Research shows that extending learning beyond classrooms enhances experiential learning, as cookbooks and take-home food education materials improve outcomes by integrating school activities into domestic environments through daily food practices [50]. This connection between school and home illustrates how experiential learning can be seamlessly incorporated across various contexts without requiring additional instruction time.

Despite these advancements, the implementation of experiential learning faces limitations. Interventions are often brief, rely on self-reported outcomes, and lack long-term follow-up, constraining conclusions regarding sustained dietary changes [14]. Furthermore, even when experiential infrastructure and curricula are available, their effectiveness is contingent on institutional support, curricular legitimacy, and educator capacity [55]. The evidence clarifies what experiential learning is not. Infrastructure, digital tools, or food exposure alone do not constitute experiential learning without intentional sensory engagement, reflection, or social mediation. Most interventions remain culturally generic, treating food as an educational object rather than a lived cultural practice [55,56].

### 3.2. Multisensory Exposure as a Mechanism of Food Learning

Multisensory exposure consolidates food-related learning because eating engages taste, smell, texture, auditory elements, and visual cues. In educational settings, multisensory exposure facilitates learning through repeated food interactions, attention to sensory attributes, and practices that convert bodily experiences into language and meaning. Evidence from school interventions with neurodivergent adolescents demonstrates the significance of these mechanisms [57]. In the BALANCE program for adolescents with Autism Spectrum Disorder (ASD), sensory exposure to foods and hands-on activities were most valued by adolescents and teachers, highlighting the need to align nutrition education with ASD’s sensory processing and cognitive rigidity characteristics [58].The study emphasizes that sensory engagement serves as an accessibility strategy, enhancing learning through visual cues and structured social experiences where students demonstrate skills [58].

Mechanistic research in sensory science has shown the importance of oral sensory exposure. Evidence shows that oral processing duration, which is influenced by food texture, affects eating behavior and intake, independent of taste. Harder textures increase chewing, reduce eating rate, extend meal duration, and decrease intake compared to softer textures, whereas sweetness intensity does not impact intake [59]. These findings suggest that multisensory learning depends on mouthfeel and oral processing, which affect satiation and food experience. This supports teaching adolescents about texture and pacing in “learning food” beyond taste preferences [57,60].

A complementary educational approach shows that multisensory exposure becomes more effective when learners externalize their sensory experiences in writing. In a Reggio Emilia-inspired food atelier, participants converted tasting experiences into drawings and narratives, showing how sensory experiences become learning objects through multiple “languages.” The study argues that sensory education improves by “democratizing” the senses—recognizing taste, smell, and touch as legitimate pathways rather than subordinates to health-focused instruction—while emphasizing the cross-modal nature of taste and the process of creating meaning through associations [61]. In practice, this indicates that multisensory exposure involves not only repeated interaction with food but also guided reflection and expression, which facilitates the conceptualization, memory, and communication of food experience.

Multisensory exposure is particularly important for selective eating and neurodevelopmental conditions in which sensory sensitivities can limit diet diversity. Evidence suggests that children with neurodevelopmental disorders show distinct weight trajectories and body composition, and taste education interventions may affect anthropometric indicators, although causal inference remains limited [62]. Reviews show that interventions for feeding difficulties use graded exposure, desensitization, and behavioral strategies, with repeated exposure enhancing acceptance of target foods while highlighting variability in protocols, outcomes, and follow-up durations [63]. These findings align with evidence that family involvement and caregiver practices determine feeding outcomes, supporting the notion that multisensory learning mechanisms are embedded in relational contexts and require alignment between educational settings and home routines [64].

The literature indicates that multisensory exposure functions through three pathways: (1) repetition and familiarity, which enhance acceptance; (2) oral processing and texture experience, which influence pacing and perceived eating properties; and (3) reflective representation, which converts sensory events into a shared meaning. For adolescent food education, these mechanisms suggest that interventions should extend beyond “telling” to structured sensory encounters—focusing on texture, utilizing visual supports, and incorporating reflection—while acknowledging that effects require continuity across settings and families [58,59,61,64].

## 4. The Teaching Taste Framework: A Multisensory Mediterranean Model

While traditional nutrition education assumes that knowledge leads to healthier food choices, evidence from studies on adolescents shows only a weak association between cognitive literacy and sustained dietary behavior [8,45]. This information-to-behavior gap reflects the structural limitations of information-based approaches, particularly in developmental contexts where attention, reward sensitivity, social belonging, and embodied learning shape what is encoded and practiced. The TASTE–MED framework addresses these limitations by reconceptualizing food literacy as an embodied and socially situated competence rather than merely accumulating nutritional information.

Information-based education often overlooks the “sensory alphabet” bottleneck: adolescents may know dietary guidelines but lack the sensory and linguistic repertoire needed to recognize and describe the recommended foods. Without experiential reference points, recommendations remain cognitively accessible but are behaviorally ineffective. TASTE–MED addresses this gap through repeated exposure, guided sensory attention, and the development of sensory vocabulary, enabling learners to articulate perceptions beyond simple liking or disliking.

Informational approaches also underestimate the social and emotional aspects of eating. Even with improved knowledge, adolescents’ choices remain shaped by habits, peer norms, and emotional associations, revealing a persistent disconnect between knowledge and action. TASTE–MED responds through relational scaffolding—shared preparation and mediated dialogue—and through the development of interoceptive awareness, supporting the recognition of hunger, satiety, and pleasure cues in food decisions.

A further limitation is the “ceiling effect,” whereby increased literacy fails to substantially modify preferences in environments dominated by ultra-processed foods that are optimized for sensory appeal. TASTE–MED shifts the pedagogical focus from knowledge transmission to competence development, proposing that food literacy emerges through experience, judgment, and participation in food practices.

Finally, informational approaches often lack contextual and cultural relevance and struggle to engage adolescents. TASTE–MED situates learning within meaningful food practices and emphasizes experiential, reflective, and socially mediated activities—tasting, cooking, and shared meals—positioning taste as the content, method, and outcome of learning. By integrating sensory foundations, social mediation, self-regulation, and cultural meaning, the framework provides a coherent basis for advancing competence-oriented food education in adolescence.

### 4.1. What Is “Teaching Taste”

Teaching Taste is an educational framework focused on developing taste competence, which is the ability to perceive, articulate, evaluate, and negotiate taste through sensory experience, reflection, and social interaction. Within this framework, taste is understood not as a fixed preference or purely biological response but as a learned, culturally mediated competence that can be intentionally cultivated through education. Teaching taste positions taste as a legitimate epistemic domain that can be learned, discussed, and questioned and refined. It integrates sensory perception with cognitive, emotional, and social processes, enabling learners to use taste as a guide for food-related decisions. This competence supports both individual choice-making and participation in shared food practices and meal communities [65,66].

### 4.2. Core Dimensions of the Teaching Taste Model

The Teaching Taste framework comprises four interrelated dimensions that support taste competence development. The sensory dimension focuses on exposure and guided attention through taste, smell, texture, and chemosensory sensations. Sensory experiences are paired with vocabulary acquisition, enabling learners to articulate their perceptions beyond simple likes or dislikes. The relational dimension recognizes taste as inherently social, with judgments shaped through commensality and interactions with peers. Shared meals and collective reflections help learners understand taste as a relational practice. The cultural dimension connects taste to identity, tradition, and local food knowledge, showing food as a cultural expression rather than a commodity. This dimension helps us understand how tastes are transmitted across generations. The reflective dimension promotes metacognition, encouraging learners to examine their preferences and how sensory, emotional, and social factors interact with them. These dimensions frame taste competence as a learnable capacity that integrates the body, mind, and society [65,67,68,69]. Sensory pedagogy develops a foundational “sensory alphabet,” enabling learners to recognize and engage with food through direct experience. Without this sensory and linguistic repertoire, dietary recommendations may remain cognitively accessible but behaviorally inoperative, as learners lack experiential references to translate their knowledge into practice.

### 4.3. The Mediterranean Diet as a Pedagogical Ecosystem

Within the TASTE–MED framework, the Mediterranean Diet is not presented as a prescriptive dietary model but as a pedagogical ecosystem in which everyday food practices may provide conditions that support taste-learning. Its structure combines sensory diversity, shared eating practices, and temporal food routines, creating conditions that facilitate repeated exposure, social participation, and contextualized food education [70].

Mediterranean food traditions typically rely on minimally processed ingredients, culinary diversity, and seasonal availability, offering repeated opportunities for adolescents to encounter varied sensory properties in meaningful contexts. These characteristics support gradual taste familiarization, linking sensory experiences to everyday cooking and eating practices rather than isolated instructional settings [70].

Equally important, Mediterranean eating practices emphasize commensality and shared food preparation, situating food experiences within relational contexts that shape taste preferences through dialogue, negotiation and participation. Eating becomes a social practice rather than an individual act, reinforcing learning processes consistent with competence-oriented pedagogies [71].

Temporal organization also plays a pedagogical role, as food consumption is structured around seasonal cycles, rituals, and recurring social events. Such temporal patterns encourage anticipation, contextual awareness, and repeated engagement with food, reinforcing sensory learning and cultural familiarity over time.

In this perspective, the Mediterranean Diet provides not only nutritional guidance but also an educational environment in which sensory exposure, social interaction, and cultural meaning converge to support the development of taste competence in daily life. Importantly, this model does not require the direct transfer of Mediterranean foods; rather, equivalent pedagogical ecosystems may be developed in other cultural contexts by preserving mechanisms such as seasonality, commensality, and sensory-rich food practices while adapting foods and culinary traditions locally [70,71].

### 4.4. Conceptual Framework of the Multisensory Mediterranean Teaching Taste Model

The TASTE–MED Model synthesizes Teaching Taste principles with the Mediterranean Diet as a pedagogical ecosystem. This framework combines sensory experience, social interaction, cultural significance, and temporal structure to develop taste competence and food literacy in youth. The model centers on experiential, reflective, and multimodal learning, engaging learners through taste, cooking, sharing meals, and dialogical reflection. Sensory engagement integrates language development, social negotiation, and guided reflection, transforming experiences into articulated knowledge. Taste serves as the content, method, and outcome of learning, following the principles of Teaching Taste. [67,68].

The model incorporates pedagogical and self-regulatory dimensions, emphasizing interoception, autonomy, and bodily awareness. Learners attend internal signals such as hunger, satiety, and pleasure, relating these to food choices and contexts. Through guided experiences, students develop an awareness of how sensory, emotional, and contextual factors influence eating behaviors. This process supports autonomy and self-regulation, enabling food decisions that are informed by external recommendations and reflective judgment.

The TASTE–MED framework does not assume that behavioral change occurs through information or willpower alone [7,14]. Instead, it proposes that knowledge translation into practice depends on two mechanisms. First, relational scaffolding through commensality and mediated dialogue enables taste judgments and food decisions to be socially negotiated within shared eating experiences to promote healthy eating. Second, self-regulatory development occurs through interoceptive calibration, as learners learn to recognize internal signals like hunger, satiety, pleasure, and discomfort regarding food choices. These processes help bridge the gap between cognitive knowledge and daily eating behaviors.

The Mediterranean Diet plays a key role in both dimensions of the model. As a sensory, social, cultural, and temporal system, it provides a coherent environment in which pedagogical and self-regulatory processes unfold. Its emphasis on seasonality, commensality, culinary diversity, and food practices creates conditions for repeated exposure, social learning, and reflection, which are essential for developing stable taste competencies. [70,71].

**Figure 1:**
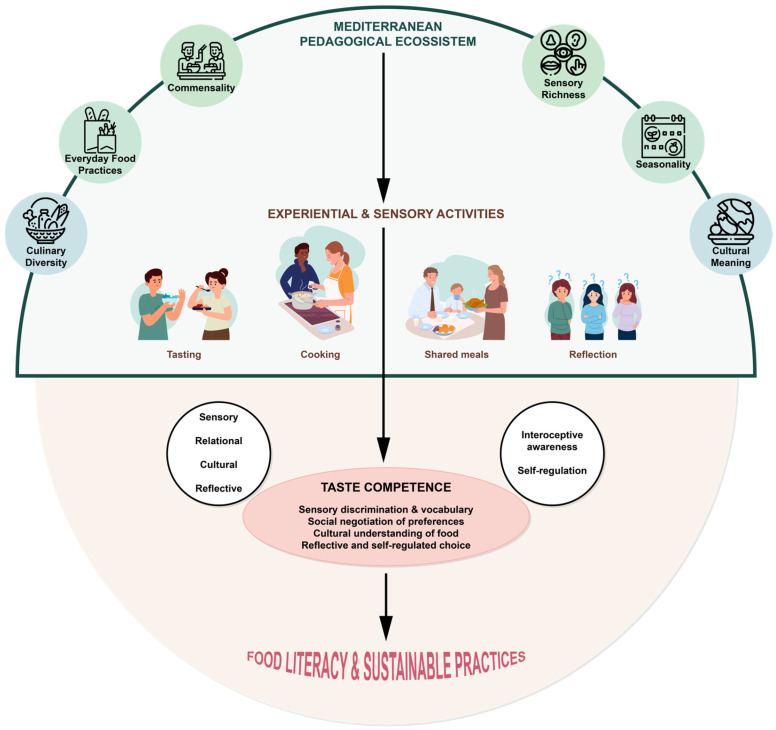
The Teaching Autonomous Sensory Taste in the Mediterranean Diet (TASTE–MED) model frames taste competence as a mediator between educational processes and food literacy outcomes. The framework integrates two dimensions: a pedagogical dimension based on experiential, reflective, and multimodal learning (including tasting, cooking, shared meals, sensory language, social interaction, and guided reflection) and a self-regulatory dimension focused on interoception, bodily awareness, autonomy, and reflective decision-making in eating behaviors. Both dimensions operate within a Mediterranean pedagogical ecosystem of sensory richness, commensality, cultural meaning, seasonality, culinary diversity and everyday food practices. Through repeated socially embedded experiences, taste competence supports food literacy development during adolescence.

The model positions taste competence as a mediator between educational experiences and food literacy outcomes. By integrating sensory perception, cultural understanding, social participation, and self-awareness, the TASTE–MED Model provides a pathway for young people to develop informed and reflective food choices. Rather than viewing food literacy as accumulated nutritional knowledge, this framework considers it as competence rooted in experiences, judgment, and shared food practices that promote sustainability. From a theoretical perspective, positioning taste competence as a mediator implies that structured multisensory and socially embedded educational experiences are expected to foster the development of taste competence, which, in turn, facilitates reflective food literacy and self-regulated eating behaviors. This conceptualization generates empirically testable propositions for future research, including whether (1) experiential Mediterranean-based pedagogies significantly enhance taste competence; (2) taste competence predicts reflective judgment and autonomy in food-related decisions; and (3) taste competence partially mediates the relationship between experiential pedagogy and food literacy outcomes. Clarifying this mediating role strengthens the theoretical positioning of TASTE–MED and supports its operationalization in longitudinal and intervention-based studies. Figure 1 illustrates the model’s structure, showing the interactions between pedagogical strategies, self-regulatory processes, and the Mediterranean pedagogical ecosystem in fostering taste competence and food literacy during adolescence.

## 5. Educational Implications and Practical Translation

### 5.1. Implications for School-Based Food Education

The TASTE–MED framework proposes shifting school-based food education from knowledge transmission to competence-oriented, experiential, and socially situated learning. Rather than emphasizing nutritional facts, the framework identifies taste competence as a fundamental educational outcome developed through sensory experiences, reflections, and interactions. This approach is supported by evidence showing that school programs with hands-on activities, cooking, inquiry-based learning, and practical food engagement yield stronger outcomes than purely didactic interventions [72,73]. To implement the TASTE–MED framework in educational contexts, it is useful to connect the main limitations of information-based food education with the pedagogical mechanisms proposed by the model. Table 1 summarizes how the constraints identified in the literature are addressed through specific dimensions of the TASTE–MED framework. By linking each limitation to corresponding pedagogical mechanisms, classroom activities, and learning indicators, the table provides an operational bridge between conceptual foundations and implementable practice, supporting educators in designing and evaluating competence-oriented food-education interventions.

From a pedagogical perspective, TASTE–MED suggests that sensory attention and sensory language should be explicitly included in the curriculum. Guided tasting, comparative sensory exploration, and systematic vocabulary development enable students to articulate, justify, and refine taste judgments, moving beyond binary likes and dislikes. This focus aligns with experiential school programs that underscore the importance of skill-building and reflective practice for food-related learning and behavior change [72,74].

The framework suggests that commensality and peer interaction can be utilized as learning mechanisms. Taste judgments are socially negotiated rather than individual, requiring shared preparation, collective eating and dialogical reflection. Whole-school and classroom interventions emphasize that social eating contexts and guided mediation are critical for embedding food education in school routines and fostering participation, rather than compliance [75,76]. Systems-based analyses corroborate that school food environments are influenced by complex networks of interacting actors, practices and policies. Consequently, isolated interventions focusing on single components are unlikely to have a sustained impact [77].

Integrating food education into the Mediterranean pedagogical ecosystem has curricular implications. Rather than a prescriptive dietary model, the Mediterranean Diet offers a structure based on seasonality, culinary diversity, and everyday food practices that support exposure and contextualized learning in the classroom. Research on whole-school food approaches indicates that such integrated, culturally grounded structures enhance feasibility, reduce fragmentation, and support sustained implementation across diverse educational contexts [75,76]. Mapping studies of school food systems highlight the importance of aligning curricular initiatives, organizational routines, and food environments. This alignment reinforces the need for pedagogical models that address systemic complexity rather than treating food education as a supplement [77].

A limitation of information-based food education is the gap between adolescents’ understanding of healthy eating and their daily food choices [8,14,45]. The TASTE–MED framework addresses this issue by integrating relational and self-regulatory mechanisms into the classroom. Relational scaffolding occurs when taste exploration and food preparation are embedded in shared experiences, allowing preferences to be negotiated through peer interaction and guided dialogue. After preparing meals in groups, students engage in moderated tasting discussions where they justify their preferences, compare experiences, and reflect on differences in acceptance, transforming food choices into shared learning processes rather than individual judgments.

Interoceptive calibration helps learners to link sensory experiences with bodily awareness. Activities such as hunger–satiety reflection diaries and guided meal reflections help students identify how hunger, fullness, pleasure, and discomfort influence their eating decisions. These reflective practices help adolescents recognize internal cues as guides for food choices, supporting autonomy and self-regulation beyond the external dietary rules.

As TASTE–MED conceptualizes taste competence as a self-regulatory and reflective capacity, schools must create opportunities for students to connect sensory experiences with bodily cues, emotions, and decision contexts. Evidence from pragmatic intervention evaluations suggests that programs are more effective when they support autonomy, reflection, and competence development rather than relying on behavior prescriptions [72,73]. Consequently, evaluation strategies should assess not only distal dietary outcomes but also intermediate indicators of taste competence, implementation fidelity, and contextual equity to fully capture the framework’s educational impact [73,74].

### 5.2. Teacher and Educator Training

Teacher capacity often limits the implementation of school-based food, nutrition, and sustainability education. Data from Australian schools show that while teachers express high perceived confidence, fewer report formal training in nutrition, food skills, or sustainability. This gap indicates the need for accessible, competency-based professional development [74]. Similarly, evidence from Finnish school leadership and teacher perspectives highlights that implementation is often inconsistent and reliant on individual teachers’ interests. Barriers such as time constraints, workload pressures, limited resources, and insufficient professional development are pervasive [76].

For the TASTE–MED initiative, training should extend beyond mere content delivery to encompass the development of a pedagogical repertoire aligned with the model’s mechanisms.

- Sensory pedagogy: guided attention, sensory vocabulary, and structured comparison tasks.
- Relational facilitation: managing commensality-based learning, peer dialogue and constructive negotiation of preferences.
- Cultural–temporal framing: using seasonality, traditions, and everyday food practices as learning resources.
- Reflective practice: encouraging metacognitive reflection on preference formation, context and decision-making.

Evidence suggests that variability in the training format and duration affects delivery fidelity. For instance, in pragmatic school programs, teacher training offered in various formats (e.g., face-to-face versus self-paced online) leads to heterogeneity in implementation quality and learning opportunities [72]. This underscores the importance of a tiered training model (core minimum plus optional advanced modules) and the provision of ready-to-use materials to alleviate teacher workload, a factor consistently identified as a facilitator in the implementation of school food education [76].

### 5.3. Family and Community Involvement

The development of taste competence and food literacy occurs across various contexts; thus, approaches confined to schools can be enhanced by incorporating families and community partnerships. Several interventions explicitly involve caregivers and community volunteers in practical activities, such as classroom cooking, utilizing observational learning and social modeling to facilitate behavior change processes [72]. Within whole-school frameworks, community partnerships are regarded as a fundamental implementation pillar, acknowledging that local food actors, families, and community organizations can extend the learning environment beyond the confines of school [75].

Nevertheless, implementation research indicates that family involvement often presents challenges in operationalization and is sometimes underdeveloped, even in feasible school models, suggesting a persistent translation gap [76]. For TASTE–MED, a pragmatic strategy involves developing low-burden, high-yield involvement options for patients.

- “Taste-at-home” micro-activities are brief, structured sensory tasks accompanied by reflection prompts.
- Family oriented recipe adaptations are linked to seasonality and cultural practices.
- Community-supported sessions, such as those involving local producers, chefs, and canteen staff, aligned with the curriculum outcomes.
- Shared events, such as seasonal Mediterranean meals or community tastings, emphasize commensality and dialogue rather than prescriptive health messages.

These strategies are consistent with the model’s relational and cultural dimensions while enhancing the ecological validity and sustainability of learning.

### 5.4. Digital Environments as Amplification Tools (Not Substitutes)

Digital tools cannot deliver smell and taste; their role in TASTE–MED is to scaffold, document, and extend the sensory experiences across settings. Rather than replacing experiential food learning, digital environments function as amplification infrastructures that support continuity and reflection across schools, homes, and communities. In TASTE–MED, digital environments serve four complementary functions aligned with pedagogical mechanisms. First, digital tools support teachers’ professional development through online microlearning modules and resources for implementing sensory facilitation routines and classroom activities. Short training modules demonstrate guided tasting sessions and peer discussions, enabling consistent implementation, despite limited formal training. Second, digital environments scaffold students’ learning through guided activities that develop sensory vocabulary without replacing embodied experiences. Students may complete digital taste journals after cooking sessions to document their sensory impressions and preference changes. Third, digital platforms strengthen home–school connections through family-facing prompts and seasonal challenges that extend classroom learning into the home. Weekly digital prompts invite students to replicate tasting activities at home and discuss them with their families, reinforcing contextual learning. Finally, digital tools assist in implementation monitoring through lightweight logs that allow educators to track activity frequency and student engagement, while supporting program adaptation across contexts.

When grounded in a clear pedagogical framework, digital infrastructures enhance feasibility and coherence while preserving embodied, relational, and culturally situated learning processes that are central to developing taste competence.

Evidence from pragmatic school-based interventions suggests that scalability in real-world settings often relies on flexible delivery support, such as online teacher training and standardized digital materials. However, this flexibility can introduce variability in implementation and outcomes if not grounded in theory [72,73]. A core principle of the TASTE–MED framework is that digital tools are most effective when serving as infrastructure for reach, coherence, and continuity, while experiential and relational learning remain central. Research on system-oriented approaches has revealed that stakeholder fragmentation impedes the successful implementation of school food interventions. This shows the importance of digital tools as infrastructure for coordination within complex school food systems [77].

The FOODWISELab digital platform supports school-based food education by providing age-appropriate, curriculum-aligned resources that scaffold sensory exploration, reflection, and social learning, rather than delivering prescriptive nutrition messages [70]. Following the TASTE–MED principles, FOODWISELab serves as a complementary learning environment that extends classroom and communal food experiences, supports teachers’ pedagogical work, and links school, family, and community contexts. Its design aligns digital tools with experiential pedagogy to avoid reducing food education to information transfer [70,74,76].

Positioning digital environments as amplification tools helps mitigate barriers to school food education, including limited instructional time, uneven access to training, and resource constraints. When grounded in a pedagogical framework, digital platforms can enhance feasibility and equity without displacing the embodied, social, and culturally situated learning central to taste competence development [72,73,76]. This is consistent with evidence that successful school food policies and interventions require coherence across multiple system levels, including pedagogy, leadership, food environment, and community engagement [77].

## 6. Limitations and Future Directions

Despite its conceptual and pedagogical strengths, the TASTE–MED framework has limitations that reflect the current evidence base and practical constraints of school-based food education research. A primary limitation of this study is the scarcity of longitudinal evidence. As recent scoping reviews of food and nutrition literacy interventions show, most programs are evaluated over short timeframes and lack follow-up assessments to determine whether the changes are sustained over time [14,78]. Similarly, research on food-related sensory activities for children indicates that no studies have examined long-term outcomes extending into adolescence or adulthood, despite the stated aim of shaping lifelong food practices [79]. Future research applying the TASTE–MED framework should prioritize longitudinal designs that examine taste competence durability and its relationship with food choices, dietary patterns, and self-regulatory capacity. Second, the measurement of sensory learning and taste competence was limited in this study. The literature shows that food education interventions rely on self-reported outcomes and non-validated instruments that capture narrow dimensions of learning, such as nutrition knowledge and cooking confidence [78]. In the domain of sensory education, outcome measures tend to focus almost exclusively on taste liking or intake, neglecting other sensory modalities, social dimensions, and reflective processes that are central to taste competence [79]. Although recent advances in food literacy measurement represent important progress, the available tools remain culturally situated and only partially capture the experiential, embodied, and relational aspects of food learning [80]. Advancing the TASTE–MED agenda will require the development and validation of multidimensional assessment tools capable of operationalizing sensory language, reflective judgment and socially mediated taste practices (Table 2).

The third limitation concerns the cultural embeddedness of Mediterranean pedagogical ecosystems. Although the Mediterranean Diet provides a coherent context for integrating sensory diversity, commensality, seasonality, and cultural meaning, its direct applicability to non-Mediterranean settings cannot be assumed. Measurement tools and educational constructs from Western or Mediterranean contexts may reflect specific food norms, values, and knowledge systems, limiting their relevance in other sociocultural environments [78,80]. Future research should investigate how the core principles of Teaching Taste can be adapted to other food cultures while preserving the sensory, relational, and reflective foundations. The implementation of TASTE–MED faces structural barriers in schools. Common challenges in food education include limited time, insufficient teacher training, lack of validated resources, and uneven institutional support [78]. Systems-based analyses further demonstrate that school food environments are shaped by complex networks of interacting actors, policies, and practices, rendering single-component or isolated interventions unlikely to succeed [77]. Within TASTE–MED, the Mediterranean Diet serves not as a prescriptive model to be replicated, but as a pedagogical ecosystem where sensory diversity, commensality, seasonality, and everyday food practices support taste learning. The framework enables the development of equivalent pedagogical ecosystems in other cultures, where local food traditions, ingredients, and practices serve similar educational functions. This “glocalized” approach preserves core pedagogical mechanisms while allowing food education to remain culturally grounded and contextually sensitive.

A critical question concerns the implementation of competence-oriented food education models in environments dominated by ultra-processed foods. Adolescents are exposed to products optimized for convenience, affordability, and sensory appeal, creating conditions that challenge healthy dietary practices among them. The TASTE–MED framework does not attempt to compete through informational persuasion alone; instead, it aims to strengthen adolescents’ capacity to navigate such environments through competence development.

Three mechanisms support the feasibility of this approach. First, developing a sensory “alphabet” through repeated exposure and guided exploration enables learners to recognize and appreciate the properties of minimally processed foods, expanding reference points beyond processed options. Second, cultivating interoceptive awareness supports self-regulation by helping adolescents connect food choices with internal signals of hunger, satiety, and pleasure, thereby reducing reliance on external consumption cues. Third, embedding food learning within social and cultural contexts—through commensality, shared preparation, and culturally meaningful food practices—provides alternative sources of belonging and rewards that can counterbalance the role of ultra-processed foods.

Feasibility depends on pedagogical mechanisms and implementation conditions. Evidence shows that isolated or short-term interventions have a limited impact. Whole-school approaches that integrate curriculum, food environments, leadership engagement, and community partnerships better support coherent food-learning experiences. Temporal continuity is essential; competence development requires repeated exposure across contexts, including connections between school and home environments.

A key limitation is that, without institutional commitment and continuity across educational settings, experiential interventions risk producing episodic effects rather than durable changes in food practice. Therefore, future research should examine long-term implementation strategies that support competence-oriented food education within complex food systems and explore mixed-methods approaches that combine behavioral observation, sensory vocabulary analysis, and self-regulation measures to empirically operationalize taste competence.

These constraints highlight the importance of embedding Teaching Taste within whole-school strategies supported by leadership, partnerships, and infrastructure. Future research should examine educational outcomes, implementation processes, cost considerations, and equity implications, in line with calls for rigorous theory-driven intervention research [78,79]. In summary, although the TASTE–MED framework offers a theoretically grounded and pedagogically integrative approach to food literacy through taste competence, its full potential depends on addressing key empirical gaps. These include the need for longitudinal designs, improved measurement of sensory and reflective learning, culturally adaptive frameworks, and systematic attention to structural constraints within educational systems (Table 1).

## 7. Conclusions

This study offers a conceptual contribution to food literacy and school-based food education by introducing Teaching Taste as a theoretically grounded framework that focuses on developing taste competence. This study proposes the TASTE–MED framework as an integrative model that reconceptualizes food education beyond information-based nutrition instruction and cooking skills training. The primary contribution articulates how Teaching Taste can be operationalized through the Mediterranean Diet, understood as a pedagogical ecosystem that is sensory-rich, socially embedded, culturally meaningful, and temporally structured. This conceptual integration provides a lens for linking embodied sensory learning, reflection, and commensality with self-regulatory capacities and shared food practices in schools to promote healthy eating. By translating theoretical principles into pedagogical dimensions and educational implications, this article addresses gaps in the literature, including the absence of theory-driven frameworks to guide the design, implementation, and evaluation of food-literacy initiatives. It positions taste competence as a legitimate educational outcome, with relevance extending beyond individual dietary behaviors to participation in the collective food culture. The TASTE–MED framework advances food literacy beyond nutritional information or skill acquisition, repositioning it as an emergent competence grounded in experience, sensory judgment, reflection and shared practices. By framing taste competence as an educational outcome, the model supports a transition from information-based nutrition instruction to competence-oriented food education, capable of fostering durable and contextually meaningful dietary practices during adolescence. Food literacy is not understood as knowledge to be transmitted but as a capability developed through embodied, relational, and culturally situated learning processes.

## Figures and Tables

**Table 1:** From limitation to mechanism: how TASTE–MED addresses key constraints of information-based food education.

Limitation in Information-Based Education	TASTE–MED Mechanism	Example Classroom Activity	Observable Indicator
Lack of sensory familiarity (“sensory alphabet” gap) prevents recognition and acceptance of recommended foods	Sensory dimension: guided exposure and development of sensory vocabulary	Guided tasting comparing textures and flavors of seasonal vegetables	Students use sensory vocabulary to describe foods beyond simple liking/disliking
Knowledge does not translate into behavior due to habitual and emotional eating patterns	Relational dimension: taste negotiated through commensality and peer interaction	Small-group cooking followed by shared meal and discussion of preferences	Students justify choices and show openness to unfamiliar foods
Self-regulation fails when based only on information and willpower	Self-regulatory dimension: development of interoceptive awareness and autonomy	Hunger–satiety reflection diary linked to meals prepared in class	Students identify internal hunger/satiety cues when discussing eating decisions
Increased literacy shows limited impact on food preferences (“ceiling effect”)	Competence-oriented experiential learning replacing information-only approaches	Repeated sensory exposure to new foods across multiple sessions	Increased willingness to taste previously rejected foods
Food education feels abstract and disconnected from lived experience	Cultural dimension: linking food to identity, seasonality, and local practices	Mapping seasonal foods and preparing culturally meaningful dishes	Students connect foods with seasonal or cultural contexts during activities
Information-based lessons fail to capture adolescent attention and motivation	Experiential and multisensory pedagogy engaging social and sensory reward systems	Classroom tasting workshops combined with peer-led evaluation	Active participation and sustained engagement during activities

**Table 2:** Key limitations of current food literacy and sensory-based education research and future research needs.

Limitation/Dimension	Central Challenge	Future Research Need
Temporal	Lack of longitudinal evidence on whether gains in taste competence and food literacy persist in late adolescence and adulthood.	Longitudinal and cohort studies examining the durability and developmental trajectories of sensory, reflective, and self-regulatory food competences.
Methodological	Heavy reliance on self-reported outcomes and narrow indicators (e.g., liking/disliking, intake frequency), with limited assessment of multisensory, social, and reflective learning processes.	Development and validation of multidimensional assessment tools capturing sensory language, embodied learning, reflection, and socially mediated taste practices.
Cultural	Cultural embeddedness of food literacy frameworks and measurement tools, particularly those rooted in Western or Mediterranean dietary norms.	Context-sensitive adaptation of Teaching Taste principles to diverse food cultures and biomes, supporting culturally responsive and “glocalized” implementations.
Systemic	Structural barriers in school settings, including rigid curricula, limited instructional time, insufficient teacher training, and fragmented food environments.	Whole-school approaches supported by integrated policies, leadership engagement, community partnerships, and system-level alignment across school food environments.

## Data Availability

The original contributions presented in this study are included in the article. Further inquiries can be directed to the corresponding author(s).
